# Quality of Douyin videos on childhood asthma: A cross-sectional content analysis

**DOI:** 10.1097/MD.0000000000051290

**Published:** 2026-10-23

**Authors:** Xiaohui, Shenggang Ding, Haihan Zheng

**Affiliations:** a Department of Pediatrics, The Third People’s Hospital of Yuhang District, Hangzhou, China; b Department of Pediatrics, The First Affiliated Hospital of Anhui Medical University, Hefei, Anhui, China; c Department of Pediatrics, The Second Affiliated Hospital of Anhui Medical University, Hefei, Anhui, China.

**Keywords:** asthma, children, Douyin, patient education, video quality

## Abstract

This study aims to evaluate the information quality of childhood asthma-related videos on the Douyin (China-market counterpart of international TikTok) short-video platform. On June 1, 2025 (Beijing time, logged-out, and non-personalized account context), we searched Douyin using the Chinese search term “儿童哮喘,” selecting the top 200 videos and ultimately including 175 videos for quality evaluation. Structured content integrity assessment tools and an instrument for judging the quality of written consumer health information on treatment choices (DISCERN) were used. The correlation between video quality and characteristics (duration, likes, comments, and shares) was analyzed. Two independent investigators performed title-and-content screening with predefined operational exclusion criteria. Inter-rater reliability was calculated before consensus adjudication. Most childhood asthma videos were created by self-identified health professionals. Content integrity scores were as follows: definition (0.325 ± 0.21), symptoms (0.521 ± 0.32), risk factors (0.294 ± 0.50), evaluation (0.425 ± 0.45), management (1.24 ± 0.47), and outcomes (0.864 ± 0.38). Among evaluated videos, 160 (91.4%) had a DISCERN score ≤50. Given that DISCERN was developed for written health information and has not been formally validated for short-form video, categorical labels (“fair,” “poor,” and “very poor”) are interpreted descriptively only. No significant differences in DISCERN dimension scores were found across 3 sorting criteria. Duration-stratified analyses within the final n = 175 dataset showed that videos with 60 to 300 seconds exhibited higher DISCERN information-reliability and overall-quality scores compared with videos ≤60 seconds; the >300 seconds stratum contained only 4 videos, and only descriptive statistics are reported for this subgroup. Top-ranked subgroup analyses were subsets sampled from the primary 175-video dataset rather than independent samples. Our analysis reveals that the quality of the sampled set of childhood-asthma-related videos retrieved from Douyin is suboptimal and lacks comprehensive information; findings cannot be generalized to all childhood-asthma-related content hosted on this platform. These findings reflect content-level deficits rather than inherent hazards of the Douyin platform.

## 1. Introduction

Asthma is a chronic inflammatory disease of the airways characterized by recurrent episodes of wheezing, breathlessness, chest tightness, and coughing, particularly at night or early in the morning.^[1]^ It is one of the most common chronic diseases in children, affecting approximately 5% to 10% of pediatric populations worldwide.^[2]^ The World Health Organization estimates that asthma causes over 339 million global cases, with a significant burden on healthcare systems due to frequent hospitalizations and emergency visits.^[3]^ Assessment of cardiorespiratory function, pulmonary function testing, and pulmonary rehabilitation are core components for long-term management of chronic pediatric respiratory diseases. In China, the prevalence of childhood asthma has increased dramatically in recent decades, rising from 1.09% in 1990 to 3.02% in 2020, largely attributed to environmental and lifestyle changes.^[4]^ Given the chronic nature of asthma, effective disease management relies heavily on patient and caregiver education to ensure proper medication adherence, trigger avoidance, and timely medical intervention. However, studies indicate that health literacy regarding asthma remains suboptimal among caregivers, leading to poor disease control and increased exacerbations.^[5]^

In the digital era, social media platforms such as Douyin (China-market counterpart of international TikTok) have become major sources of health information, particularly for younger demographics. Douyin has more than 600 million daily active users in China.^[6]^ Douyin serves as a critical channel for disseminating health-related content, including asthma management advice.

Despite its widespread use, concerns persist regarding the quality and accuracy of health information on social media. Previous studies on platforms like YouTube and Facebook have revealed a significant proportion of misleading or low-quality medical content.^[7]^ However, limited research has evaluated asthma-related information on short-video platforms like Douyin, where engaging yet potentially inaccurate content may influence health behaviors. This study assesses the reliability, quality, and comprehensiveness of childhood asthma content on Douyin, to clarify content quality issues rather than claiming platform-level risks.

## 2. Methodology

### 2.1. Search strategy

On June 1, 2025 (Beijing Time, using a logged-out, non-personalized Douyin account), videos were collected using the Chinese search term “儿童哮喘.” To minimize algorithm-driven personalization effects: no user login, no prior browsing history on the test device, device geolocation set to mainland China, and the app version was the latest official domestic Douyin release. Douyin offers 3 sorting options: “overall ranking,” “most recent,” and “most liked.” The top 200 videos were selected, consistent with prior research indicating users typically view 100 to 300 videos. This single-keyword search may have omitted relevant content indexed under alternative terms.^[8,9]^

The top-200 videos were loaded and captured sequentially under Douyin default comprehensive-ranking algorithm by scrolling the result page. Three built-in sorting modes (default comprehensive ranking, most-liked, and most-recent) generate distinct ranked video lists. We did not repeat the identical search on multiple dates to assess temporal stability of search ranking, which constitutes a limitation of this cross-sectional snapshot design.

Inclusion and operational exclusion criteria: Inclusion: Short-video content discussing childhood asthma in the Chinese language. Exclusion: Irrelevant content: Videos with a primary theme unrelated to childhood asthma, only brief incidental mention of asthma. Duplicate video: Identical or near-identical video clips re-uploaded by different accounts. Advertisement: Explicit commercial promotion for drugs, medical institutions, or healthcare products.

Two investigators independently screened retrieved videos against the above criteria. Disagreements were resolved via consensus discussion or 3rd-investigator arbitration.

Coder-assigned uploader-source operational definitions. First, general user: ordinary Douyin accounts without any self-stated medical-related professional background in their public profile. Second, self-identified health professionals: accounts whose public profile explicitly declares them as practicing physicians, nurses, or other clinical healthcare providers. Different clinical professions were grouped into this single category. Important: we did not independently verify licenses, qualifications, or real-world identity for these accounts. Third, news agencies: formal institutional news-service organizations. Fourth, individual science communicators: independent noninstitutional accounts creating popular-science content without declared clinical-healthcare-provider identity.

### 2.2. Video quality assessment tools

Content completeness evaluation: **T**he content-completeness coding framework was adapted from Goobie et al,^[10,11]^ and modified to fit the childhood-asthma context based on official Chinese pediatric asthma clinical guidelines. The adapted scoring criteria were jointly developed by 2 senior pediatric respiratory clinicians (coauthors SD and HZ). The 6 evaluation domains (disease definition, symptoms, risk factors, assessment, management, and outcomes) were retained from the original published framework; granular, item-level scoring rules were iteratively discussed and finalized by these 2 clinicians. Content validity of the adapted instrument was evaluated via expert review against national pediatric-asthma guideline recommendations. Crucially, the complete coding framework presented in Table S1, Supplemental Digital Content 1 was fully locked and finalized before any study-video assessment began. Every included video was evaluated applying this identical full scoring scheme with no post hoc adjustment of criteria. Each dimension was rated on a scale from 0 to 2: 0 (absence of content), 0.5 (minimal content), 1 (partial content), 1.5 (substantial content), and 2 (comprehensive content). Full detailed scoring items are provided in Supplementary Table S1, Supplemental Digital Content 1 for transparency.

Assessment of information-reliability and treatment-quality: The DISCERN instrument^[12]^ was employed for this purpose. Notably, the DISCERN instrument was originally developed and validated for written consumer health information; it has not undergone formal psychometric validation for short-form video media. In this study, DISCERN serves only as an exploratory assessment tool. Its derived categorical labels (very poor, poor, fair, good, and excellent) are used purely for descriptive grouping and do not represent validated gold-standard quality benchmarks for short-video content. This tool consists of 16 items categorized into 3 dimensions: dimension 1 (items 1–8) evaluates reliability, dimension 2 (items 9–15) addresses the quality of treatment information, and dimension 3 (item 16) assesses overall quality. Each item receives a score ranging from 1 to 5, culminating in a total DISCERN score between 16 and 80. The total scores are classified as follows: very poor (16–26), poor (27–38), fair (39–50), good (51–62), and excellent (63–80).

Assessment procedure: Two clinical experts, each possessing over a decade of clinical experience, conducted the video quality assessments. Both evaluators independently reviewed the videos and applied the content completeness and DISCERN assessment tools. Inter-rater reliability before consensus adjudication was calculated using intraclass correlation coefficients (ICC): content completeness ICC = 0.82, 95% confidence interval: 0.76 to 0.88; DISCERN scoring ICC = 0.85, 95% confidence interval: 0.80 to 0.90, indicating excellent inter-rater consistency. Final scores were established through consensus discussion. In instances of disagreement between rater 1 and rater 2, an arbitrator (rater 3) was engaged to make the conclusive decision. Consensus was achieved for all final scores.

Analysis based on sorting: Utilizing Douyin sorting capabilities, we performed 2 sets of subgroup analyses. Top-30 videos retrieved under 3 sorting modes (default comprehensive sorting, most-liked, and most-recent), all extracted strictly from the final included primary dataset (n = 175); overlapping videos across subgroups may exist. Duration-stratified analyses were performed using all eligible videos within the final n = 175 dataset, with strata defined as <60 seconds (n = 32), 60 to 300 seconds (n = 139), and >300 seconds (n = 4). This analytic approach focused on videos with high potential exposure and user interaction.^[13]^

### 2.3. Statistical analysis

Statistical analyses were conducted utilizing SPSS version 26.0 (IBM Corporation) for descriptive statistics and comparative analyses, while figures were produced with GraphPad Prism version 9.5.1 (GraphPad Software, Inc.). Normality of distributions was formally tested prior to selecting parametric or nonparametric tests. Data that adhered to a normal distribution were reported as mean ± standard deviation, and analyses of differences were carried out employing 1-way analysis of variance (ANOVA), followed by post hoc testing via the Bonferroni correction method. Conversely, data that did not follow a normal distribution were expressed as median (*P*_25_, *P*_75_), with differences assessed using the Kruskal–Wallis test, complemented by Dunn multiple comparisons for post hoc analysis.^[14]^

Homogeneity of variances was assessed using Levene test for datasets submitted to 1-way ANOVA. When normality assumption or homogeneity-of-variance assumption was violated, Welch-corrected ANOVA or nonparametric Kruskal–Wallis test was applied as alternative accordingly. For small-size subgroups, Kruskal–Wallis sensitivity analysis outputs are reported within result tables rather than only mentioned in methods. To evaluate the relationships between quantitative variables, Spearman rank correlation analysis was implemented.^[15]^

Multiple-testing strategy: The prespecified a priori primary outcomes were DISCERN total score and overall composite content-completeness score. All remaining analyses including individual content-dimension comparisons, DISCERN subdimension analyses, subgroup comparisons stratified by uploader type, publication year, video duration, sorting criteria, and all Spearman correlation analyses were designated exploratory analyses. Bonferroni correction was applied exclusively for post hoc pairwise comparisons corresponding to primary outcomes only, to control the family-wise error rate. We did not implement additional multiplicity correction for the large set of exploratory statistical tests; accordingly, all exploratory results should be interpreted considering the potential risk of type-I error inflation. A statistical significance threshold α = 0.05 was applied for primary outcome analyses.

### 2.4. Ethics statement

This study analyzed publicly accessible short-video content on Douyin. Our institutional review board granted an ethics exemption for this study, as no identifiable personal creator information (username, avatar, and private profile data) was collected or stored; only publicly visible video metadata and content were extracted for aggregate analysis.

## 3. Results

A total of 200 videos related to childhood asthma were collected from the short-video platform Douyin. After excluding 12 videos irrelevant to the topic, 10 duplicate videos, and 3 advertisements, 175 videos were ultimately included for evaluation. The screening process is illustrated in Figure 1. As of the data collection date, the videos collectively garnered 10,450,238 likes, 365,421 comments, and 2,584,126 shares. Detailed information on video uploads by year and view counts is presented in Table 1.

**Table 1:** Overall characteristics of the videos (n = 175).

Characteristics	Videos, n (%)
Time (s)	
<60	32 (18.3)
60–300	139 (79.4)
>300	4 (2.3)
Years since upload	
2022	41 (23.4)
2023	48 (27.4)
2024	52 (29.7)
2025	34 (19.4)
Aggregated engagement metrics	
Likes	10,450,238
Comments	365,421
Shares	2,584,126

Percentages were calculated based on the total primary sample of 175 Douyin videos (Douyin: China-market counterpart of international TikTok). Time-stratified counts represent all eligible videos in the full dataset. Aggregated metrics refer to cumulative total likes, comments and shares across all included videos.

**Figure 1.**
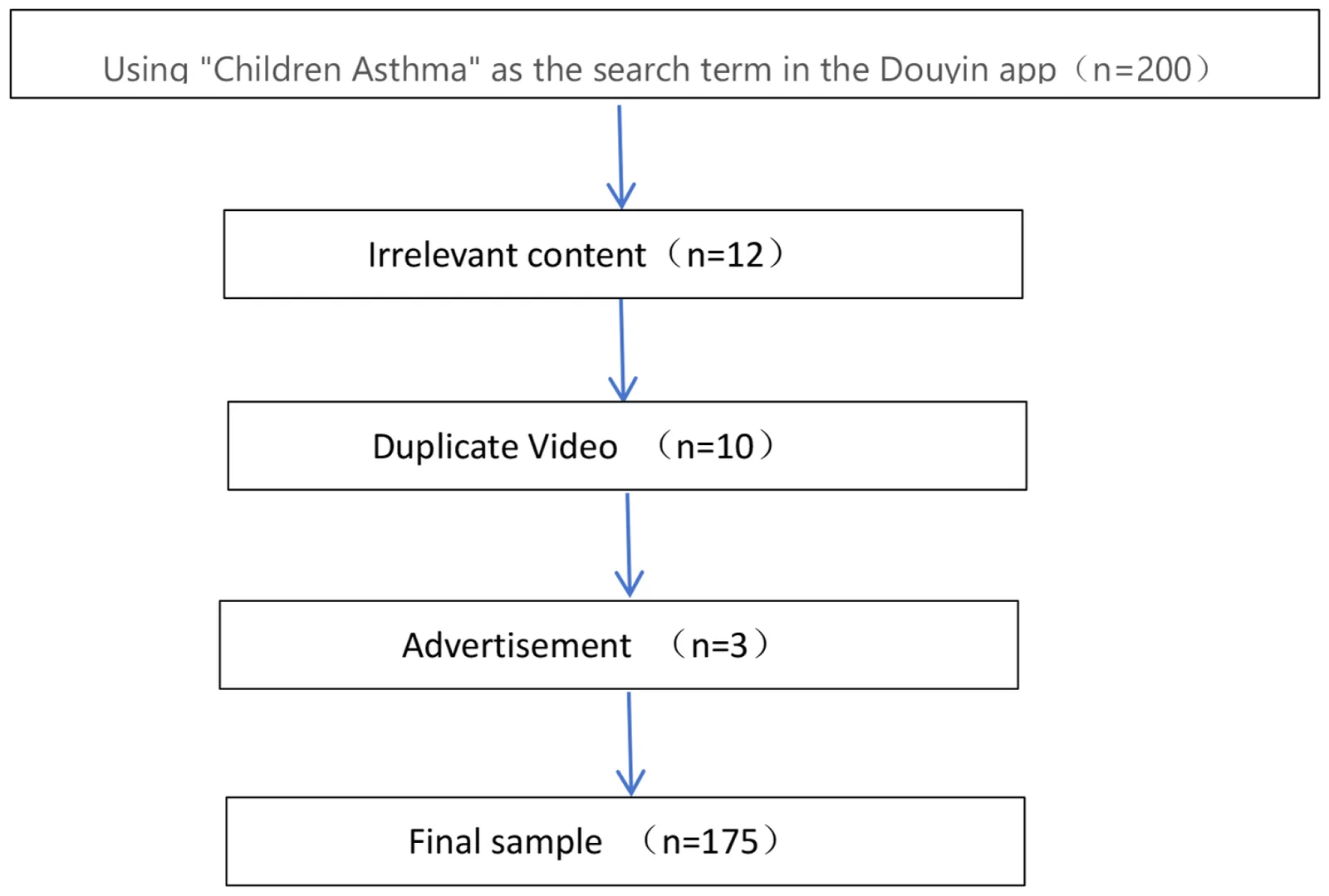
Flowchart for retrieval and screening of childhood-asthma-related videos from Douyin.

The Douyin platform predominantly featured videos related to childhood asthma, with a significant majority (73.1%) produced by self-identified health professionals. This was followed by contributions from general users (21.7%), individual science communicators (2.9%), and news organizations (2.3%), as illustrated in Table 2. Credentials for self-identified health professionals were not independently verified in this study. In our analysis, the duration of the videos varied, ranging from 15 to 442 seconds. Notably, videos created by news agencies had the longest duration, followed by those from self-identified health professionals, general users, and individual science communicators, as detailed in Table 3.

**Table 2:** Characteristics of the sources of childhood asthma-related Douyin videos (N = 175).

Source	Description	Videos, n (%)
General user	Ordinary Douyin users without self-declared medical background	38 (21.7)
Self-identified health professionals	Accounts self-reporting as healthcare providers (e.g., physicians and nurses); credentials were not independently verified by the study team	128 (73.1)
News agencies	Formal news-service organizations	4 (2.3)
Individual science communicators	Independent individuals producing popular-science content (e.g., popular-science writers)	5 (2.9)

Percentages calculated on total primary sample n = 175 Douyin videos. “Self-identified health professionals” denotes self-declared account status; no external credential verification was performed in this study. Douyin: China-market counterpart of international TikTok.

**Table 3:** Characteristics of childhood asthma-related Douyin videos, by source.

Variables	Likes, median (IQR)	Comments, median (IQR)	Shares, median (IQR)	Days since upload (d), median (IQR)	Duration (s), median (IQR)
General user	1625 (623–3768)	725 (189–1623)	212 (89–324)	446 (258–640)	45 (28–76)
News agencies	242 (58–795)	62 (12–248)	43 (4–124)	568 (196–812)	102 (64–218)
Self-identified health professionals	642 (453–865)	215 (54–564)	163 (32–295)	421 (256–608)	53 (46–62)
Individual science communicators	16,400 (985–42,612)	3245 (642–4128)	1954 (242–8632)	785 (365–1125)	36 (25–186)

All values presented as median (interquartile range). Data are derived from the primary sample of n = 175 Douyin videos. Health-professional status is self-reported and not externally verified. Douyin: China-market counterpart of international TikTok.

IQR = interquartile range.

A radar chart visually displays the scores of childhood asthma-related videos across 6 dimensions: definition (0.325 ± 0.21), symptoms (0.521 ± 0.32), risk factors (0.294 ± 0.50), evaluation (0.425 ± 0.45), management (1.24 ± 0.47), and outcomes (0.864 ± 0.38) (Fig. 2). Statistical analysis revealed a significant difference in symptom dimension scores across different years, with 2025 scores being significantly higher than those in 2022 (*P* < .05) (Table 4). These are exploratory analyses without multiplicity correction.

**Table 4:** Comparison of content completeness scores by year (scores $\bar{x}\pm \;s$).

	2022	2023	2024	2025	*F*	*P*
Definition	0.29 ± 0.12	0.32 ± 0.16	0.34 ± 0.18	0.35 ± 0.24	0.184	.909
Symptoms	0.47 ± 0.25	0.46 ± 0.30	0.44 ± 0.36	0.55 ± 0.35*	4.102	.008
Risk factor	0.31 ± 0.59	0.26 ± 0.45	0.31 ± 0.48	0.30 ± 0.50	0.090	.964
Evaluation	0.40 ± 0.25	0.36 ± 0.41	0.65 ± 0.44	0.62 ± 0.45	2.121	.100
Management	1.20 ± 0.55	1.12 ± 0.43	1.30 ± 0.44	1.29 ± 0.45	0.330	.804
Outcomes	0.64 ± 0.67	0.82 ± 0.51	0.90 ± 0.60	0.88 ± 0.55	0.412	.748

$\bar{x}+s$, mean ± standard deviation. Analyses were performed on n = 175 Douyin videos. Normality was tested prior to 1-way analysis of variance; Kruskal–Wallis test was additionally applied as sensitivity analysis when normality assumptions were violated. Prespecified primary outcomes and exploratory subgroup analyses were distinguished to handle multiple-comparison inflation. Douyin: China-market counterpart of international TikTok.

* : Symptom scores in 2025 were significantly higher than those in 2022.

**Figure 2.**
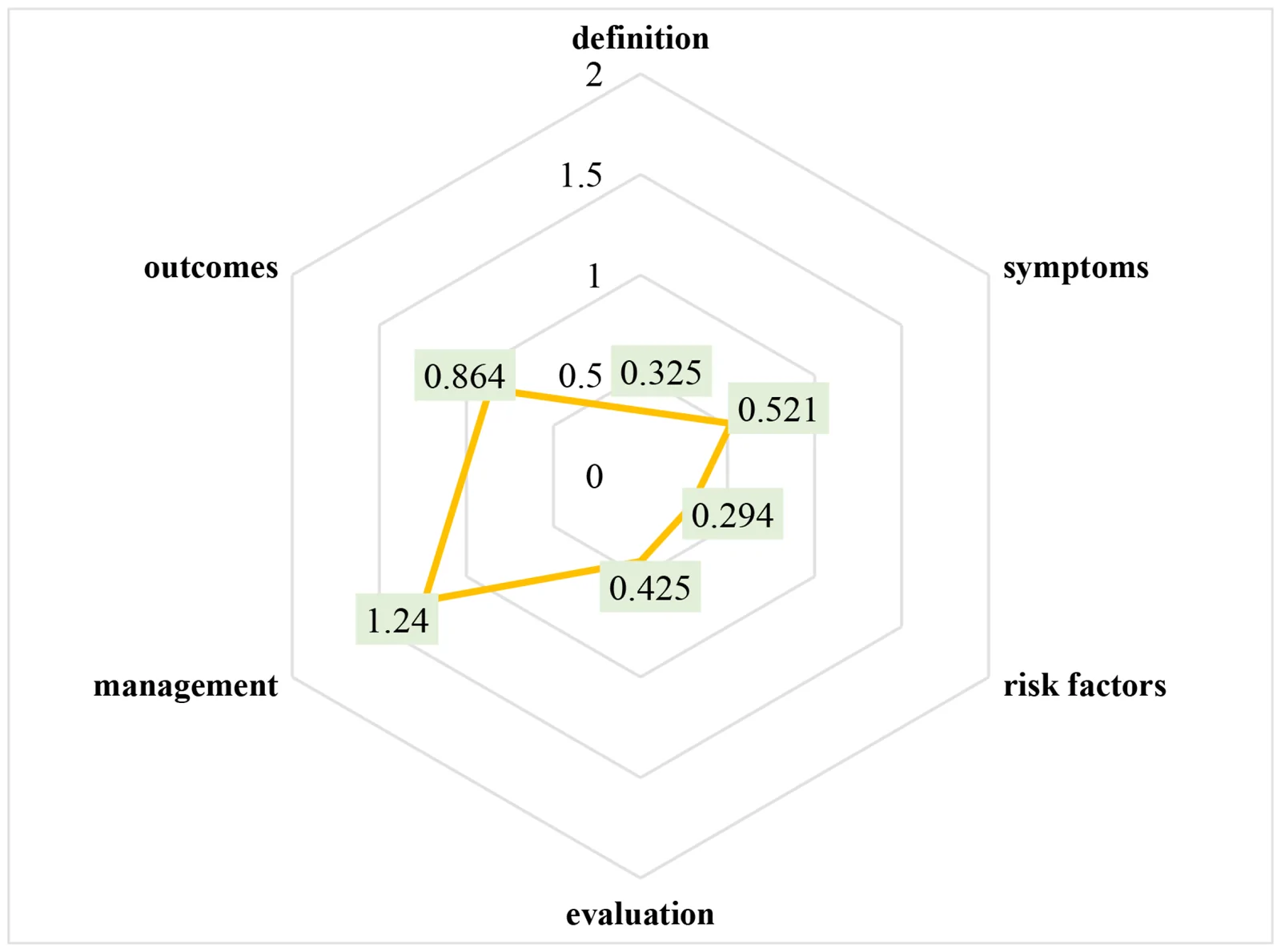
Radar chart illustrating content-completeness dimension scores for childhood-asthma-related Douyin videos (n = 175).

Among the evaluated videos, 160 (91.4%) had a DISCERN score ≤50. For descriptive grouping according to DISCERN original cutoffs: 70 videos (40%) fell into the “fair” range, 77 videos (44%) into “poor,” 13 videos (7.4%) into “very poor,” and only 15 videos (8.6%) fell within the “good” score range. We stress these categorical labels are exploratory and are not validated grading standards for short-video health content. No statistically significant difference was observed in DISCERN scores among videos from different uploaders (*P* > .05) (Table 5). These are exploratory analyses without multiplicity correction.

**Table 5:** DISCERN scores by different types of uploaders [scores, *M*(*P*_25_, *P*_75_)].

Source of videos	General user (38)	Self-identified health professionals (128)	Individual science communicators (5)	News agencies (4)	*H*	*P*
Reliability of the videos	23.6 (20.5, 24.2)	23.5 (21.0, 24.4)	21.0 (13.5, 28.0)	21.0 (18.0, 22.5)	2.952	.229
Quality of treatment choices	12.5 (9.5, 15.5)	14.0 (11.0, 17.0)	11.0 (8.0, 14.5)	18.0 (14.5, 21.0)	5.555	.062
Overall quality	2.4 (2.2, 2.6)	2.0 (1.8, 3.0)	2.0 (1.7, 2.6)	2.0 (1.2, 2.5)	1.163	.559
Total score	38.5 (34, 43.5)	39.0 (34.0, 44.0)	34 (26.0, 45.5)	39.5 (38.0, 43.5)	3.047	.218

*M*(*P*_25_, *P*_75_): median (25th, 75th percentile). *H*: *H* statistic from Kruskal–Wallis test. Analyses were performed on primary sample n = 175 Douyin videos. Inter-rater reliability before consensus adjudication: content-completeness ICC = 0.82 (95% CI 0.76–0.88); DISCERN ICC = 0.85 (95% CI 0.80–0.90). Self-identified health-professional status was not independently verified. Douyin: China-market counterpart of international TikTok.

CI = confidence interval, DISCERN = an instrument for judging the quality of written consumer health information on treatment choices, ICC= intraclass correlation coefficients.

No statistically significant differences were observed in DISCERN dimension scores across the 3 video sorting criteria (*P* > .05). Duration-stratified descriptive results for the final n = 175 dataset are shown in Table 6. Among the 2 larger strata (<60 and 60–300 seconds), videos with duration 60 to 300 seconds showed higher DISCERN information-reliability and overall-quality scores compared with videos <60 seconds (Table 6 and Fig. 3).

**Table 6:** Comparison of scores by different ranking and duration filter criteria (scores $\bar{x}\pm \;s$).

Ranking methods	Reliability of the videos	Quality of treatment choices	Overall quality	Total score
Default sorting (n = 30)	23.10 ± 4.82	13.70 ± 4.49	2.73 ± 0.79	39.53 ± 8.81
Most-liked (n = 30)	25.43 ± 6.40	14.60 ± 5.41	2.80 ± 0.85	42.83 ± 10.90
Newest (n = 30)	23.60 ± 4.46	15.00 ± 6.24	2.63 ± 0.93	41.23 ± 13.21
*F*	1.133	0.452	0.289	0.661
*P*	.327	.638	.750	.520
Duration-filtered subsets (all videos within final primary dataset n = 175)				
<60 s (n = 32)	19.83 ± 4.01	11.97 ± 3.58	1.87 ± 0.57	33.67 ± 6.45
60–300 s (n = 139)	22.77 ± 4.38	13.13 ± 3.59	2.40 ± 0.50	38.30 ± 5.58
>300 s (n = 4)	27.95 ± 6.62	19.40 ± 5.91	3.25 ± 0.71	50.60 ± 12.14

Subgroup analyses are restricted strictly to final included primary dataset n = 175. The >300 seconds stratum includes only 4 videos; hence, only descriptive statistics are provided without inferential statistical testing (no *F* statistic and *P* value calculated for duration-stratified comparison). Analyses for 3 ranking modes (default sorting, most-liked, newest) were performed on top-30 subsets extracted from n = 175 dataset. $\bar{x}\pm s$, mean ± standard deviation. Normality assumptions were tested; Kruskal–Wallis test was used as sensitivity analysis for small subgroups. Douyin: China-market counterpart of international TikTok. No inferential statistics for >300 seconds group due to very small sample size.

**Figure 3.**
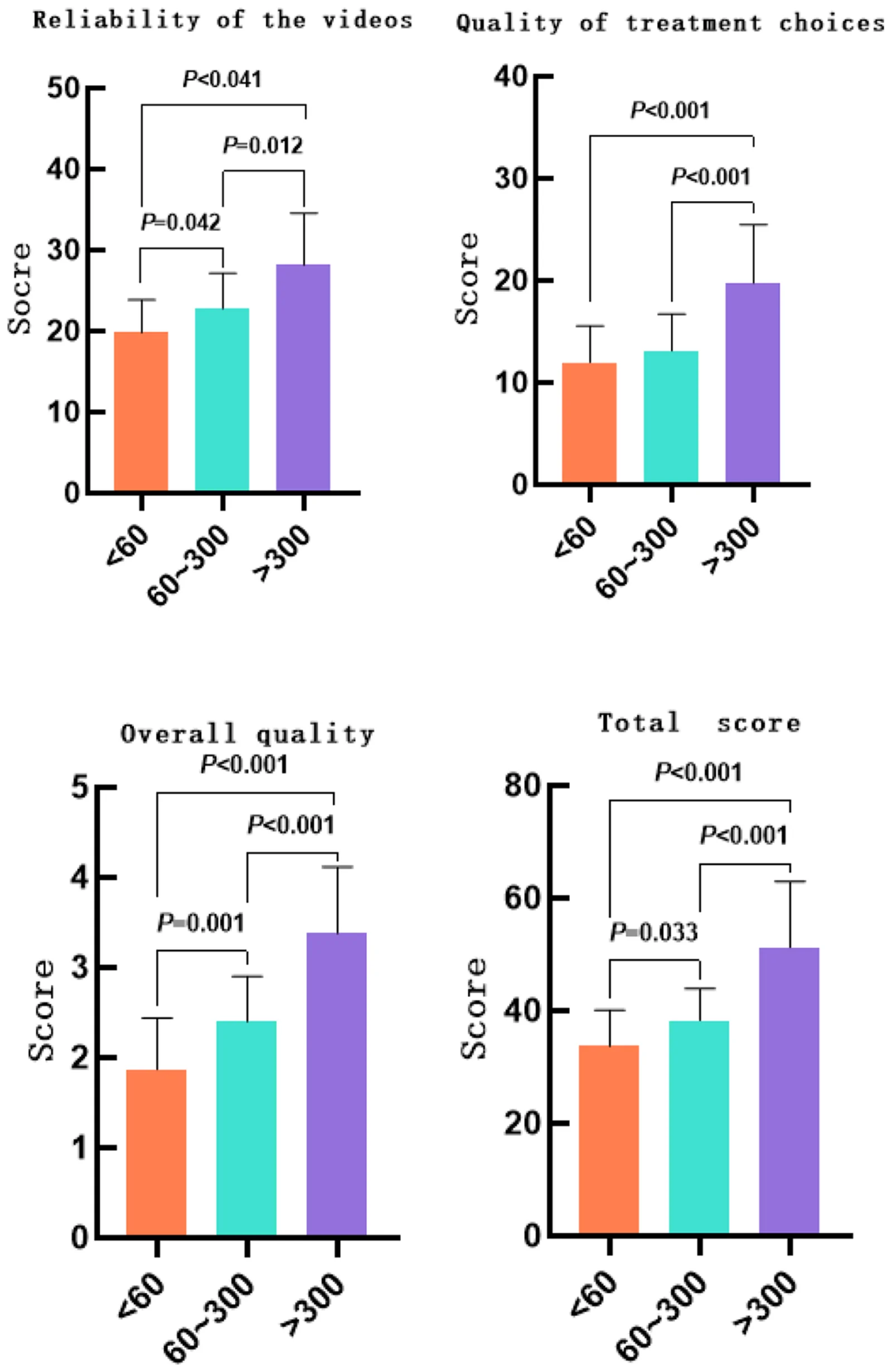
Comparative results of DISCERN dimensions for the top-ranked videos of different durations. DISCERN = an instrument for judging the quality of written consumer health information on treatment choices.

Correlation analysis: The Spearman correlation analysis indicated significant positive associations among several variables: likes and comments (*r* = 0.904, *P* < .001), likes and shares (*r* = 0.841, *P* < .001). Days since upload exhibited statistically significant negative Spearman correlations with likes, comments, and shares (Table 7). This represents an observed cross-sectional association only; it cannot establish that newer videos intrinsically generate higher engagement, given that older videos accumulate views and interaction over longer time windows and platform-algorithm exposure may change over time. Additionally, positive correlations were found between comments and shares (*r* = 0.707, *P* < .001). Notably, no statistically significant correlation was detected between video duration and popularity-related metrics including likes (*r* = 0.051, *P* = .502), comments (*r* = 0.071, *P* = .348), or shares (*r* = 0.033, *P* = .660, see Table 7 and Fig. 4). These are exploratory analyses without multiplicity correction.

**Table 7:** Analysis of the relationship between video variables and DISCERN scores *r (P*).

	Total score	Likes	Comments	Shares	Days since upload	Duration (s)
Total score	1					
Likes	0.141 (.061)	1				
Comments	0.100 (.185)	0.904 (<.001)	1			
Shares	0.077 (.307)	0.841 (<.001)	0.707 (<.001)	1		
Days since upload	−0.227 (.002)	−0.221 (.003)	−0.175 (.020)	−0.192 (.010)	1	
Duration	0.409 (<.001)	0.051 (.502)	0.071 (.348)	0.033 (.660)	−0.140 (.063)	1

Spearman rank-order correlation analysis was performed on the primary n = 175 Douyin videos. *r* = Spearman correlation coefficient. Douyin: China-market counterpart of international TikTok.

**Figure 4.**
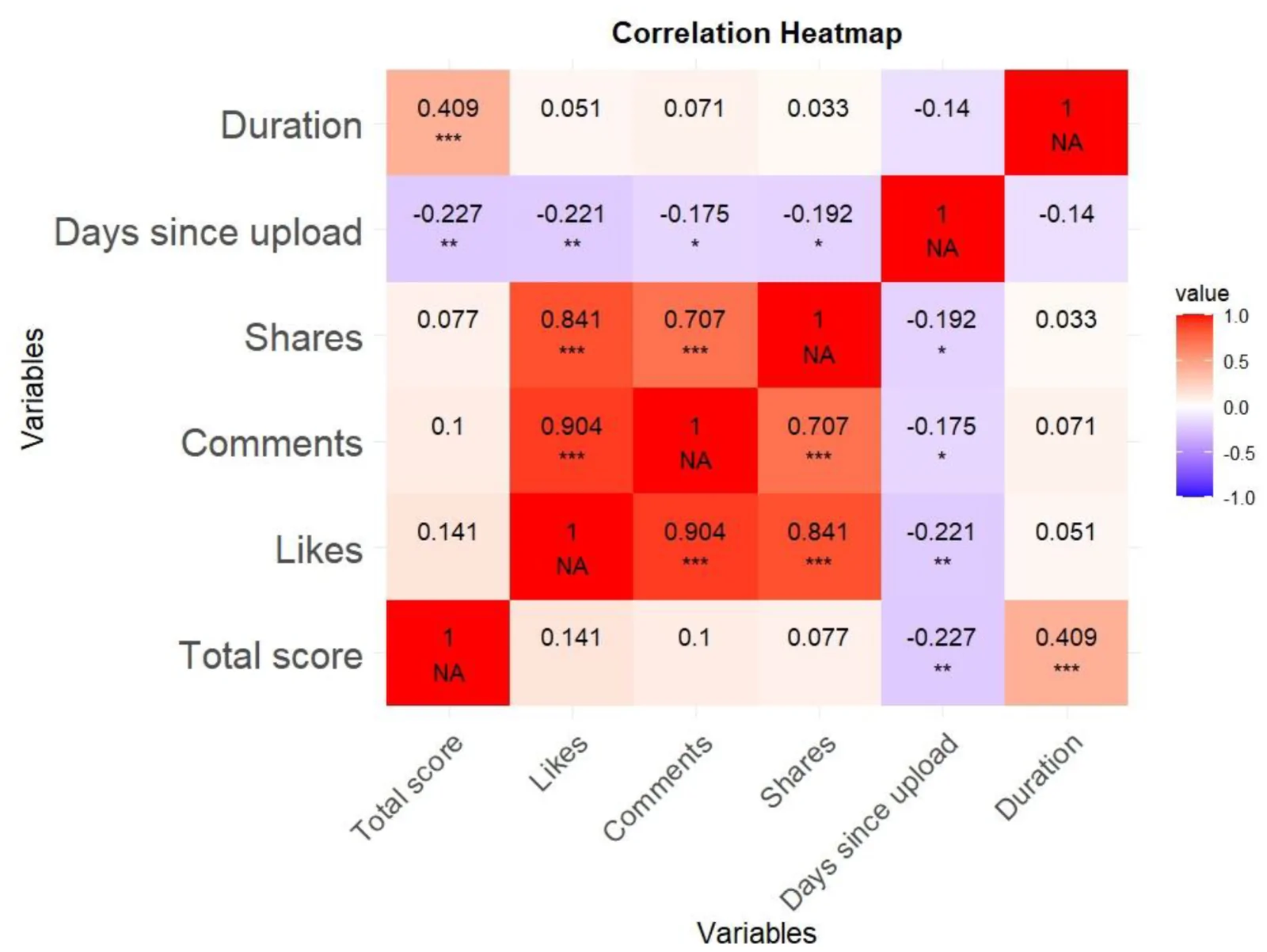
Heatmap of Spearman correlation between video variables and DISCERN total score for childhood-asthma-related Douyin videos (n = 175). DISCERN = an instrument for judging the quality of written consumer health information on treatment choices.

## 4. Discussion

### 4.1. Principal findings

This cross-sectional investigation examined the attributes of videos concerning childhood asthma on Douyin at a designated moment and assessed their quality through the application of the DISCERN instrument. A significant portion of childhood-asthma-related videos were uploaded by accounts labeled as self-identified health professionals; such labeling may enhance viewers’ perceived credibility of content, yet these self-reported professional identities were not externally validated in our study. Nevertheless, the evaluation based on DISCERN scores revealed that the quality of the content produced by these accounts fell short of anticipated standards. Importantly, it is worth mentioning that Douyin does not verify the credentials of users who purport to be physicians, which raises concerns regarding potential identity misrepresentation, such as individuals posing as doctors to enhance their video viewership.

### 4.2. Factors influencing video popularity

The number of likes, comments, and shares reflects, to some extent, the popularity of a video or post.^[16,17]^ We found positive correlations among likes, comments, and shares, indicating that popular videos are more likely to receive comments and shares. The number of comments was also positively correlated with the number of shares, suggesting that videos with more comments are more likely to be shared. We observed cross-sectional negative associations between days since upload and engagement metrics. No strong correlation was observed between video quality and popularity metrics, an important implication for platform governance.

### 4.3. Overall video quality

Our findings indicate that the general quality of short videos related to children’s asthma on Douyin is inadequate. This observation is consistent with assessments of other health-related video materials available on the platform. We attribute this inadequacy to the insufficient enforcement of content review mechanisms on short-video platforms and the lack of regulations concerning the types of content creators, which may detrimentally affect the quality of the material presented. Prior research has demonstrated that there is not necessarily a direct correlation between the quality of videos and their popularity.^[18,19]^ Hence, it is essential for short-video platforms to enhance the oversight of health communication content to ensure the scientific integrity of the information being disseminated.^[9]^

Moreover, our investigation uncovered that the content within these videos often lacked balance. More than half of the videos did not adequately address critical topics such as definitions, symptoms, risk factors, and assessments. This may stem from the intrinsic constraints of short videos, which limit the ability to thoroughly explore various topics within a brief duration, thus resulting in diminished completeness scores. Prior studies have indicated that the thoroughness of video content has a positive relationship with its popularity.^[8,20,21]^ Consequently, we advocate that relevant videos should effectively clarify definitions, broaden their content, and provide updated treatment information to improve knowledge completeness and fulfill users’ requirements for comprehensive health information.

## 5. Limitations and future research

This study has notable limitations. Our sampling depended on a single Chinese search term and a single data-collection date; we captured only top-ranked search results. Therefore, our findings describe properties of this specific sampled snapshot rather than the complete population of childhood-asthma-related videos on the Douyin platform. Although we adopted a logged-out account and standardized device settings to minimize algorithm-driven personalization, time-dependent and location-related residual ranking bias cannot be fully excluded in Douyin search outputs.

The >300-second video stratum in our final dataset had a very small sample size (n = 4). We assessed content completeness and quality but did not directly measure misinformation or clinically harmful advice. The application of DISCERN to short-form video is exploratory; categorical quality labels derived from DISCERN cutoffs are not formally validated for this media format. This study assessed content completeness and exploratory DISCERN quality metrics; we did not systematically quantify the frequency of factual misinformation, detect specific harmful clinical recommendations, measure real-world patient-level asthma outcomes, or user health-behavior changes. Therefore, we cannot draw direct conclusions regarding clinical harm induced by these videos.

Our evaluation was confined to short videos available on Douyin, necessitating further research into the quality of information on alternative platforms. In addition, due to the dynamic and algorithm-driven nature of Douyin search results, the outcomes may fluctuate based on the timing of the search and algorithm personalization. We only selected top-ranked videos, which may introduce selection bias; uploader professional credentials could not be independently verified; subjective rating may exist despite high inter-rater ICC; a cross-sectional snapshot cannot capture evolving online content; some subgroups have small sample sizes; findings are limited to Chinese-language domestic Douyin and cannot generalize to other countries or international TikTok. The sample size was limited to 200 videos, which may not capture a comprehensive overview, and this study exclusively focused on short videos in the Chinese language, while videos in other languages have yet to be assessed. Future studies should further evaluate the accuracy and clinical safety of specific asthma recommendations on short-video platforms.

## 6. Conclusion

Our analysis reveals that the quality of sampled videos pertaining to children’s asthma on Douyin is subpar and lacks pertinent information. These results indicate content-level deficits rather than inherent danger or hazards of the Douyin platform. Parents, caregivers, patients, and other users should critically evaluate childhood asthma-related content when using Douyin for health information.

## Author contributions

**Formal analysis:** Haihan Zheng.

**Funding acquisition:** Haihan Zheng.

**Methodology:** Shenggang Ding.

**Project administration:** Xiaohui, Shenggang Ding.

**Resources:** Xiaohui, Shenggang Ding.

**Software:** Xiaohui.

**Supervision:** Xiaohui, Shenggang Ding, Haihan Zheng.

**Validation:** Xiaohui, Shenggang Ding.

**Visualization:** Xiaohui.

**Writing – original draft:** Xiaohui, Shenggang Ding.

**Writing – review & editing:** Haihan Zheng.


