# Supplementary material for: Quality of Douyin videos on childhood asthma: A cross-sectional content analysis
Source: Medicine (Baltimore). 2026 Oct 23;105(43):e51290. doi: 10.1097/MD.0000000000051290 (PMC13706433; doi:10.1097/MD.0000000000051290)
Supplement: Supplementary file 1 [file medi-105-e51290-s001.docx]

Table S1. Modified DISCERN benchmark criteria.

| Score* | Reliability Score |
| --- | --- |
| 1 score | Is the video clear, concise, and understandable? |
| 1 score | Are reliable sources of information used? (i.e., publication cited, speaker is specialist) |
| 1 score | Is the information presented balanced and unbiased? |
| 1 score | Are additional sources of information listed for patient reference? |
| 1 score | Are areas of uncertainty/controversy mentioned? |

*The criteria of each aspect were scored separately, and 1 point is given for every Yes and 0 points for No.
